# Complete Genome Assembly of Myxococcus xanthus Strain DZ2 Using Long High-Fidelity (HiFi) Reads Generated with PacBio Technology

**DOI:** 10.1128/MRA.00530-21

**Published:** 2021-07-15

**Authors:** Rikesh Jain, Bianca H. Habermann, Tâm Mignot

**Affiliations:** a Aix-Marseille Université, CNRS, Institut de Microbiologie de la Méditerranée (UMR 7283), Turing Center for Living Systems, Marseille, France; b Aix-Marseille Université, CNRS, Institut de Biologie du Développement de Marseille (UMR 7288), Turing Center for Living Systems, Marseille, France; Montana State University

## Abstract

Myxococcus xanthus is a Gram-negative social bacterium belonging to the order *Myxococcales* of the class Deltaproteobacteria. It is a facultative social predator found in soils across the globe and is thought to be crucial for the microbial ecosystem. Here, we report a complete high-quality reference genome of the M. xanthus strain DZ2.

## ANNOUNCEMENT

Myxococcus xanthus, a social bacterium, preys on a broad range of microbes, including bacteria as well as fungi (1, 2). Its predatory behavior involves moving or gliding (socially and individually) into prey colonies, killing prey cells that it comes in contact with and consuming them for nutrition (3, 4). The predatory behaviors of M. xanthus have been researched for many decades; however, the mechanisms of how it recognizes and kills the diverse preys are not clearly understood yet. Interestingly, different strains of M. xanthus show different predation efficiencies against the same prey (1, 2). Therefore, comparing the genomes of these strains might help in identifying key genes, pathways, and molecules involved in prey recognition and killing. It is for this purpose that we sequenced, assembled, and annotated the complete genome sequence of our lab strain, DZ2, which was originally procured from David R. Zusman’s lab (5).

DZ2 cells were grown at 32°C in CYE (Casitone yeast extract) medium, and genomic DNA was extracted using a MasterPure DNA purification kit (catalog [cat.] number 85200) (Epicentre/Lucigen, Middleton, WI) following the manufacturer’s protocol. The high-fidelity (HiFi) sequencing library was prepared using the SMRTbell express template prep kit (PacBio, CA), and genome sequencing was performed using single-molecule real-time (SMRT) sequencing on the PacBio Sequel II system at the DRESDEN-concept Genome Center, Germany. A total of 98,235 subreads were generated with a mean subread length of 6,019 bp, totaling 591 Mb, which represents approximately 60-fold coverage. Circular consensus reads were called using Code Composer Studio (CCS) version 5 (PacBio) on the raw PacBio subreads, and the resulting consensus reads with a quality value (QV) of >20 or >99% accuracy were used to assemble the genome with HiCanu version 2.1 (6). This resulted in a single circular contig of 9,359,382 bp (approximately 220 kb larger than the DK1622 genome) with a GC content of 68.86%. Genome annotation was performed using the NCBI Prokaryotic Genome Annotation Pipeline (PGAP) version 5.1 (7), which predicted a total of 7,408 protein-coding sequences (CDSs), 12 rRNA genes, 66 tRNA genes, 4 noncoding RNA (ncRNA) genes, 86 pseudogenes, and 4 CRISPR arrays in the DZ2 genome. Default parameters were used for all software unless otherwise noted.

A previous version of the DZ2 genome sequence (8) existed as a draft assembly with 87 contigs. It is around 88 kb shorter than our PacBio complete assembly, suggesting large coverage gaps and possibly missing important loci. It was sequenced with 454 GS-FLX Titanium technology, a second-generation sequencing technology, which generates much shorter reads (average read length, 450 bp) than the third-generation sequencing platform PacBio Sequel II (average read length, 10,000 bp). Highly accurate (99.8%) long high-fidelity PacBio reads enabled the resolving of large repetitive and low-complexity genomic regions, thus closing all the gaps, a task which remains challenging with short-read sequencing (9).

Uninterrupted and accurate genome sequence information is crucial for many downstream genomic analyses, such as biosynthetic gene cluster mining and gene synteny comparison, as well as for understanding genome plasticity, the patterns of transposon distribution, and gene expression regulation.

### Data availability.

The raw sequencing data can be found at the NCBI SRA database under the accession number PRJNA701418. The complete genome sequence has been deposited in GenBank under accession number CP070500.
